# Porcine congenital splayleg is characterised by muscle fibre atrophy associated with relative rise in *MAFbx *and fall in *P311 *expression

**DOI:** 10.1186/1746-6148-2-23

**Published:** 2006-07-25

**Authors:** Peck-Toung Ooi, Nuno da Costa, Julia Edgar, Kin-Chow Chang

**Affiliations:** 1 Molecular Medicine Laboratory, Division of Animal Production and Public Health, Faculty of Veterinary Medicine, University of Glasgow, Glasgow, UK

## Abstract

**Background:**

Porcine congenital splayleg (PCS) is the most important congenital condition of piglets, associated with lameness and immobility, of unknown aetiology and pathogenesis, hence the need to better understand the condition by defining, in the first instance, its histopathology and molecular pathology.

**Results:**

*Semitendinosus, longissimus dorsi*, and *gastrocnemius *muscles were removed from 4 sets of 2-day-old splayleg piglets, each with a corresponding normal litter mate. Based on immunohistochemistry and histological image analysis, PCS piglets showed significantly smaller fibre size without any accompanying sign of inflammation. Although there was no dramatic change in fibre type composition in affected muscles, several structural myosin heavy chain genes were significantly down-regulated. *MAFbx*, a major atrophy marker, was highly up-regulated in nearly all PCS muscles, in comparison with controls from normal litter mates. In contrast, *P311*, a novel 8 kDa protein, was relatively down-regulated in all the PCS muscles. To investigate a functional role of *P311 *in skeletal muscle, its full-length cDNA was over-expressed in murine C2C12 muscle cells, which resulted in enhanced cell proliferation with reduced myotube formation. Hence, reduced *P311 *expression in PCS piglets might contribute to atrophy through reduced muscle cell proliferation. *P311*, predictably, was down-regulated by the over-expression of calcineurin, a key signalling factor of muscle differentiation.

**Conclusion:**

We demonstrated that PCS is a condition characterised by extensive fibre atrophy and raised fibre density, and propose that the combined differential expression of *MAFbx *and *P311 *is of potential in the diagnosis of subclinical PCS.

## Background

Porcine congenital splayleg (PCS), also known as straddlers, and myofibrillar hypoplasia, is a clinical condition of newborn piglets, characterised by muscle weakness, resulting in the inability to properly stand and walk, with affected limbs extended sideways or forwards [1]. It is arguably the most important congenital defect of commercial piglets and causes significant economic loss to pig farmers [2]. The prevalence of PCS can range from less than 1% in most farms to over 8% in some establishments [3].

PCS can be found at or a few hours after birth in up to 2 to 3 piglets in an affected litter. Arising from long periods of recumbency, abrasions and ulceration may develop, which could predispose the affected individual to secondary arthritis, pododermatitis, and osteomyelitis of the digits. Mortality rate can reach 50% due to starvation or crushing by the sow. Affected piglets can recover after a week if supportive treatment is provided. PCS is prevalent in Landrace, Large White breed, and in other heavily muscular breeds [4-6]. Male and female piglets appear susceptible to PCS in similar proportion [1,5,6]. There are, however, conflicting reports on the association of PCS with litter size or birth weight [5,7].

PCS appears to be a multifactorial condition of unknown aetiology. The predisposing factors are thought to include genetics and environmental factors, such like nutrition, management, pharmacological administration, and mycotoxins. Pregnant sows treated with glucocorticoids [8], and experimentally fed *Fusarium *(F-2) toxin (zearalenone) contaminated grain [9] showed higher incidence of PCS. However, histological changes in glucocorticoid induced or mycotoxin-induced PCS are distinct from naturally occurring PCS [10]. The role of dietary choline appears unimportant in the prevention of PCS [11,12]. Administration of pyrimethamine, an antibiotic often used in combination with a sulphonamide, to pregnant Goettingen minipigs [13], and of prostaglandins to induce parturition in late pregnancy [14] have been reported to lead to higher incidence of PCS.

A range of pathological lesions has been described for PCS, the most common feature being the presence of myofibrillar hypoplasia, often interpreted as an immaturity of the muscle [1,10]. Myofibrillar hypoplasia ranges from a slight reduction of myofibrillar content to severe myofibrillar deficiency, with vacuolation, focal degeneration and necrosis. However, myofibrillar hypoplasia is not exclusive to PCS as the condition is also found in clinically normal piglets. The descriptive finding of myofibrillar hypoplasia is therefore not be diagnostic of PCS [10]. Furthermore, subjective microscopic and ultrastructural muscle examination had found no significant qualitative changes between normal and PCS piglets [3,15]. Presently, there is a lack of objective morphological information available on PCS muscles. The term hypoplasia refers to underdevelopment of a tissue or organ that results in a decrease in cell number. Atrophy refers to a decrease in the size of a tissue or organ caused by disease or disuse. It is not certain if clinical PCS is related to a reduction of fibre size (atrophy), number (hypoplasia) or both. Additionally, no histochemical, biochemical, or molecular changes have been reported that are characteristic of PCS. Therefore, there is a need to better define the histopathological, biochemical and molecular changes that take place in muscles of PCS. The development of objective cellular and molecular parameters to assess clinical PCS could add valuable information to our understanding of the pathogenesis of the disease. With recent advances in imaging technology and quantitative PCR, morphological analysis and relative muscle gene expression can now be conducted with greater precision. We describe here the histopathological and molecular characterisation of PCS. We found that PCS piglets had smaller fibre size and higher fibre density in the *semitendinosus *(ST), *longissimus dorsi *(LD) and *gastrocnemius *(G) muscles. *Muscle Atrophy F-box *(*MAFbx*), a marker of muscle atrophy, was more highly expressed in all PCS muscles. Conversely, expression of *P311*, a novel 8 kDa protein with a conserved PEST domain, was down-regulated in all PCS muscles tested.

## Results

### Muscle fibre atrophy in PCS muscles

**Figure 1:**
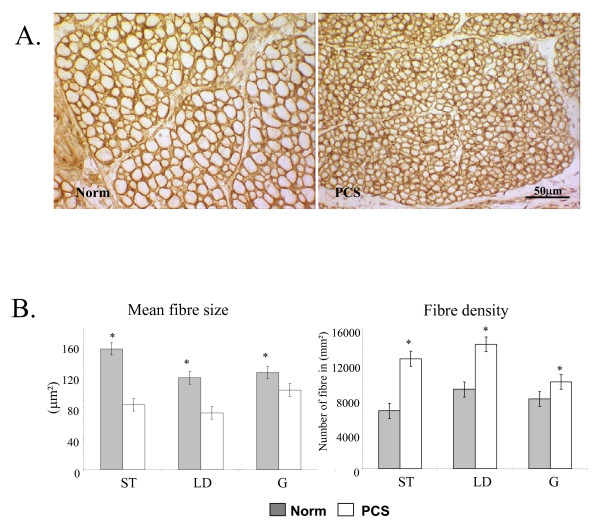
**Disparity of fibre size and density between PCS piglets and their normal litter mates**. ST, LD and G muscles were removed from 4 sets of 2-day-old splayleg male piglets, each comprised an affected and a normal litter mate. Muscles were immunostained for dystroglycan, myosin heavy chain (MYH) slow and MYH fast. For each muscle sample, 6 microscopic fields were randomly selected for morphometric analysis. (**A**) Typical muscle fields of normal and PCS (ST muscles) immunostained for dystroglycan. PCS fibres were clearly smaller than those in the normal counterpart. (**B**) Fibre cross-sectional area was determined in PCS and normal muscles. PCS piglets showed smaller fibre size and higher fibre density in all three muscles. Differences between pair-wise combinations of the least square means were tested for significance (*p < 0.05). Asterisks indicated statistical significance between normal and PCS muscle. The error bars indicate standard error.

**Figure 2:**
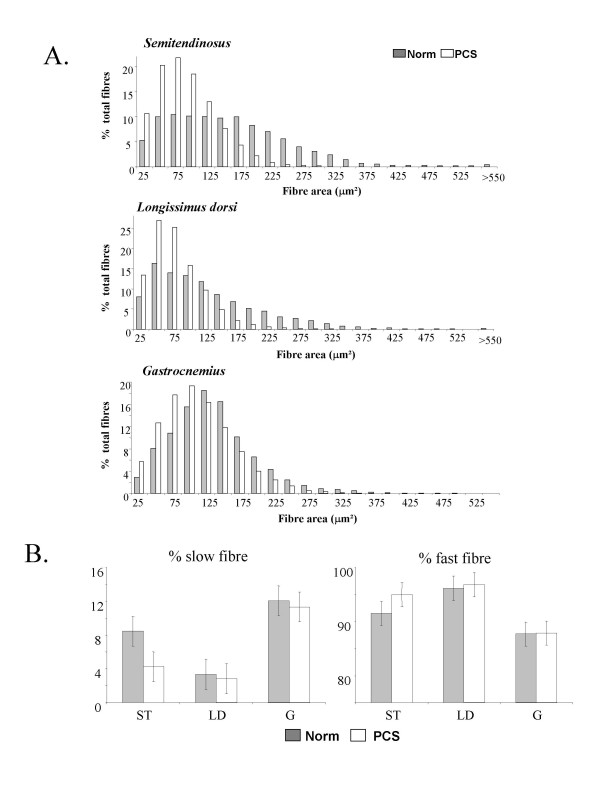
**Distribution of muscle fibre cross-sectional areas in four sets of 2-day-old PCS piglets, each with a corresponding normal litter mate**. At least 10,000 fibres were measured per muscle section and grouped by size in 25 μm^2 ^increments (**A**). ST and LD muscles of PCS piglets showed a narrow and steep pattern of distribution. In ST muscles, more than 50% of PCS fibres were less than 100 μm^2^, compared with normal fibres where only 35% were less than 100 μm^2^. In LD muscles, more than 50% of PCS fibres were less than 75 μm^2^; compared with normal fibres only about 35% were less than 75 μm^2^. G muscles of PCS piglets showed a similar but smaller fibre size distribution than their normal litter mates. (**B**) However, no significant difference in slow or fast fibre type composition was found between normal and affected muscles. The error bars indicate standard error. Norm = normal; PCS = porcine congenital splayleg.

Morphological and fibre type analyses were conducted on 4 sets of muscles from 4 separate litters of pigs (3 muscles per pig: ST, LD and G); each set comprised one 2-day-old affected PCS piglet and a normal litter mate. Histological examination of all PCS muscles showed no evidence of significant inflammatory change (data not shown). The average fibre cross-sectional area of affected PCS piglets was consistently smaller than their normal counterparts in all 3 muscles (Fig. 1). Consequently, fibre density (fibre number per unit area) from affected individuals was higher in the 3 muscles (Fig. 1B). PCS piglets showed higher distribution of fibre number in the smaller fibre range than normal piglets (Fig. 2A). The contrast in fibre size distribution was particularly evident in the ST and LD of PCS piglets, which showed a narrow and steep distribution pattern of smaller fibres (Fig. 2A). Hence fibre atrophy was a feature of PCS muscles. However, based on immunostaining for myosin heavy chain (MYH) slow and MYH fast fibres, no significant difference was found in fibre type composition between normal and affected muscles (Fig. 2B).

### Selective reduction of MYH expression in PCS muscles

**Figure 3:**
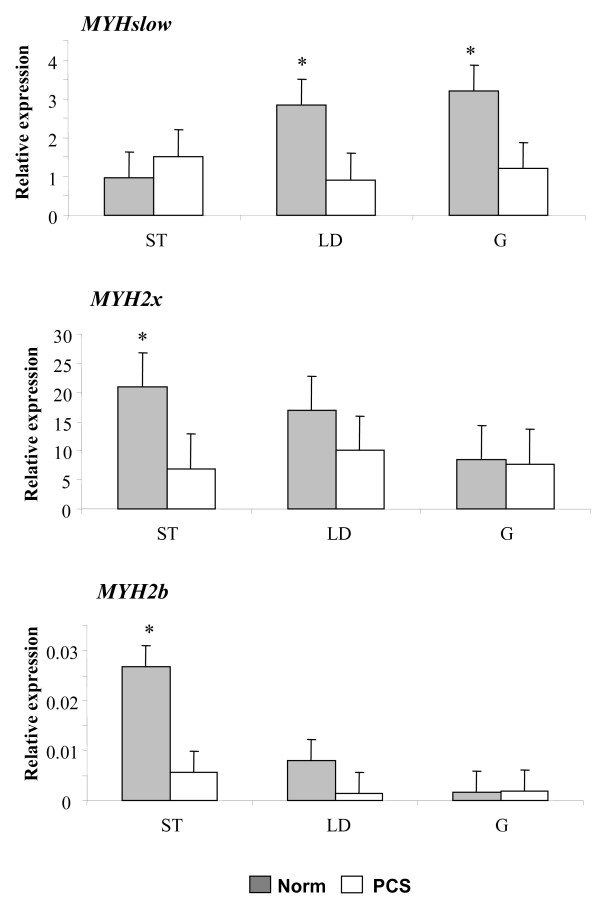
**Comparison of muscle gene expression between PCS affected and normal muscles**. Quantitative expression of *MYHslow*, *MYH2x *and *MYH2b *in ST, LD and G muscles, presented as combined results of 4 sets of 2-day-old PCS piglets. Differences between pair-wise combinations of the least square means were tested for significance (*p < 0.05). PCS piglets showed significantly lower expression of *MYHslow *in LD and G muscles, and lower expression of *MYH2x *and *MYH2b *in ST muscles. Asterisks indicate statistical significance between normal and PCS muscles. Error bars indicate standard error.

To further assess the phenotypic differences between muscles of normal and PCS piglets, TaqMan quantitative real-time PCR was performed on the muscles from the 4 sets of litter mates to determine the relative mRNA expression levels of *MYHembryonic, MYHperinatal, MYHslow*, *MYH2a*, *MYH2x*, *MYH2b *and α-*actin*. PCS piglets showed significantly lower expression of *MYHslow *in LD and G muscles (Fig. 3). Expression of *MYH2x *and *MYH2b *in ST muscle was also statistically lower in PCS piglets (Fig. 3). No significant difference was found between normal and PCS piglets for *MYHembryonic*, *MYHperinatal*, *MYH2a *and α *-actin *(data not shown).

### Up-regulation of MAFbx and down-regulation of P311 in PCS muscles

**Figure 4:**
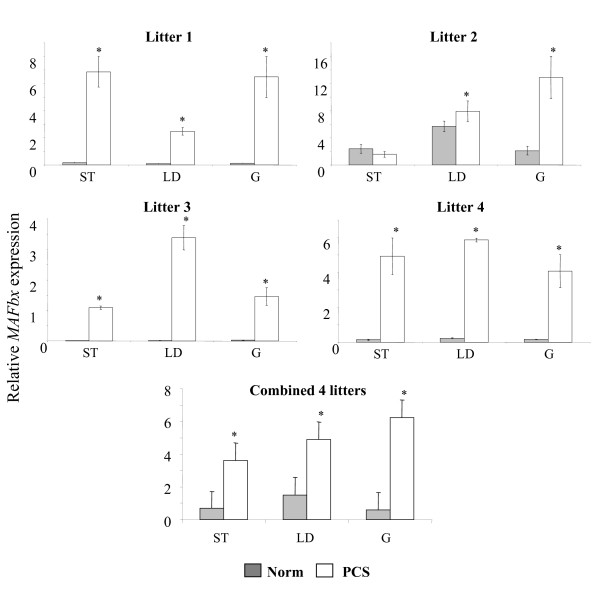
**Quantitative PCR was performed for *MAFbx *expression**. With the exception of ST muscles in litter 2, *MAFbx *was more highly expressed in all PCS muscle samples compared with the corresponding normal muscles. Results of each litter are expressed as mean ± standard deviation from triplicate samples within the same experiment. Combined results of 4 sets of PCS piglets also tested for significance (*p < 0.05) in differences between pair-wise combinations of the least square means. Norm = normal; PCS = porcine congenital splayleg.

**Figure 5:**
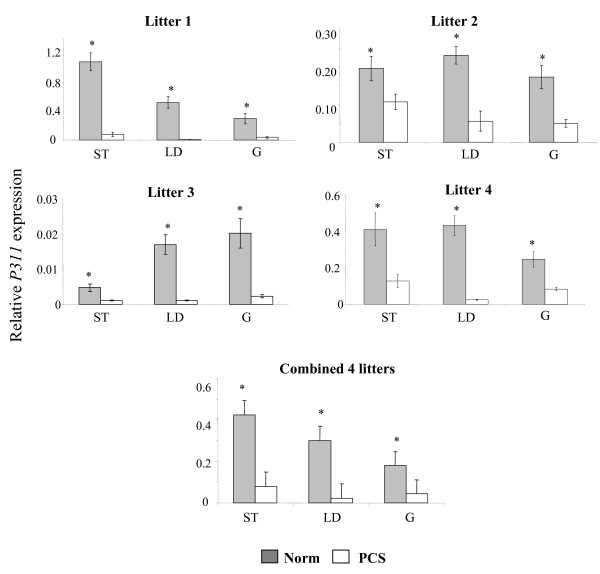
**Quantitative PCR was performed for *P311 *expression**. In contrast to *MAFbx*, *P311 *expression was down-regulated in all PCS muscle samples. Results of each litter are expressed as mean ± standard deviation from triplicate samples within the same experiment. Combined results of 4 sets of PCS piglets also tested for significance (*p < 0.05) in differences between pair-wise combinations of the least square means. Norm = normal; PCS = porcine congenital splayleg.

The ubiquitin-proteasome proteolysis pathway is a principal route of muscle atrophy in which MAFbx, an E3 ubiquitin ligase, was found to be consistently up-regulated in a variety of muscle atrophic conditions [16,17]. Because of the close association of *MAFbx *expression with muscle atrophy, we determined the relative expression of porcine *MAFbx *in normal and PCS muscles (Fig. 4). We found that in almost all muscle samples examined, with the exception of ST muscle in litter 2, *MAFbx *expression was clearly up-regulated in PCS piglets (Fig. 4). Another growth-related gene associated with muscle atrophy, *P311*, is a novel gene reported to be down-regulated in a number of muscle atrophic conditions [17] but up-regulated under moderate dietary restriction [18]. In contrast to *MAFbx*, *P311 *expression in PCS muscles was consistently down-regulated in comparison with normal controls (Fig. 5). It is worthy to note that the significant differences in *MAFbx *and *P311 *expression between normal and affected muscles of litter 2 were not as great as in the other 3 litters, which might be a reflection of differing gradation of the disease process (Fig. 4 and 5). Moreover, the wide variation in expression levels of *MAFbx *and *P311 *between litters, were not entirely surprising. Endogenous gene expression is known to vary widely between individual pigs of the same genetic background and age, reared under identical husbandry conditions [19].

**Figure 6:**
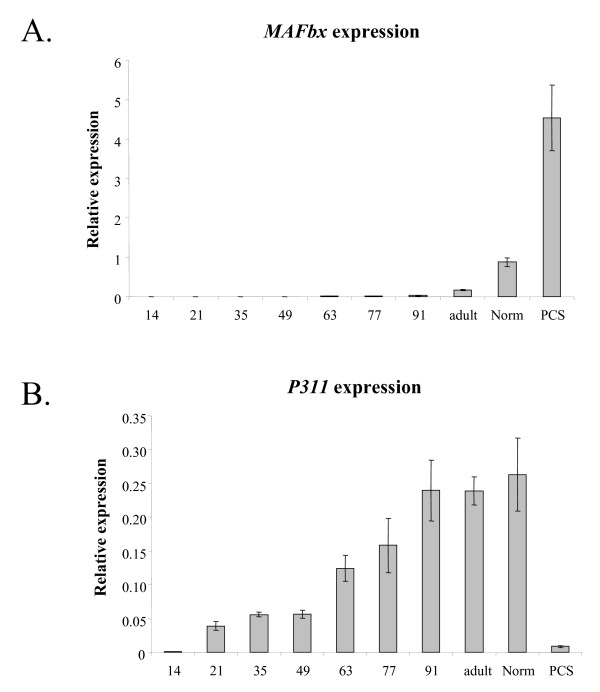
**Developmental expression of *MAFbx *and *P311 ***. Expression of (**A**) *MAFbx *and (**B**) *P311 *at different stages of embryonic/foetal development (14, 21, 35, 49, 63, 77 and 91 days of gestation), in 24-week-old (adult) pigs, in 2-day-old PCS piglets and corresponding normal litter mates (Norm) were determined. Embryonic/foetal and adult cDNAs were pooled samples from 3 individuals. PCS and Norm cDNAs were pooled from the LD muscles of the 4 sets of litter mates. Expression of *MAFbx *was detected at basal levels throughout gestation and into adulthood but was elevated in PCS muscles. *P311 *expression showed rising levels throughout development. However, its expression in PCS piglets was much reduced. Results expressed as mean ± standard deviation from triplicate samples within the same experiment. Norm = normal; PCS = porcine congenital splayleg.

As little is known about the normal developmental expression pattern of *MAFbx *and *P311*, we examined their embryonic and muscle foetal expression at different stages of gestation, and their expression in adult muscle (Fig. 6). Real-time PCR results showed that *MAFbx *expression remained at basal levels throughout gestation and in adult muscle (Fig. 6A). In relation to normal muscles, *MAFbx *expression in PCS LD muscles was significantly elevated (Fig. 6A). Real-time PCR results showed that *P311 *expression in normal animals increased with gestation and remained raised in newborn and adult muscles. However, the expression of *P311 *in 2-day-old PCS piglets was considerably down-regulated (Fig. 6B).

### P311 over-expression in C2C12 cells by adenovirus infection and stable transfection

**Figure 7:**
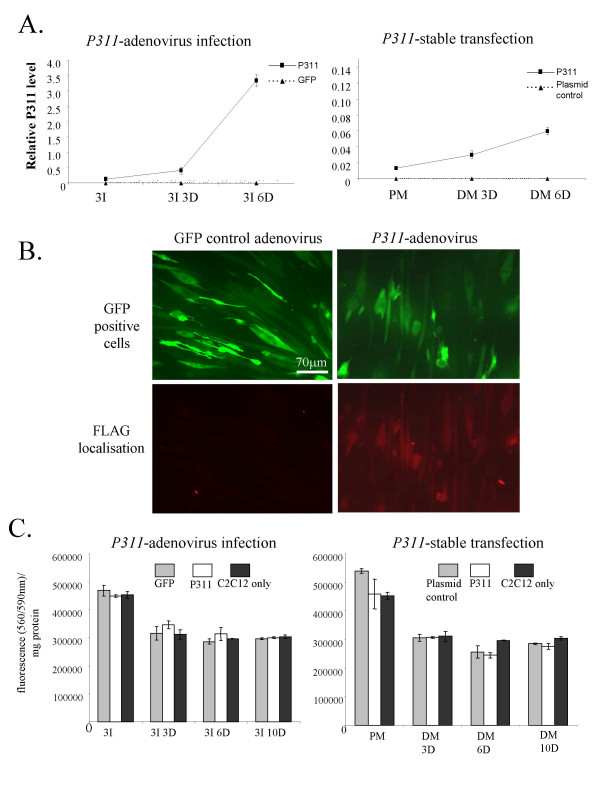
**Over-expression of *P311 *in C2C12 cells**. (**A**) *P311 *expression, through infection with an adenoviral construct and through stable transfection with an expression plasmid, was determined over a time course. Proliferating cells were infected for 3 days in proliferation medium [PM] (3I), followed by 3 days in differentiation medium [DM] (3I 3D) or 6 days in DM (3I 6D). Stably transfected cells were incubated for 3 days in PM (PM), followed by 3 days in DM (DM 3D) or 6 days in DM (DM 6D). Results expressed as mean ± standard deviation from triplicate samples within the same experiment. (**B**) Adenovirus-mediated (at 0.5 MOI) expression of *P311 *in C2C12 cells (infected for 3 days during proliferation followed by 6 days of differentiation), viewed under fluorescence for GFP and immunostained for FLAG. *P311*-infected cells showed co-localisation of both proteins. (**C**) The viability of *P311-*infected and *P311*-transfected C2C12 cells was similar to control cells over an extended culture period, as demonstrated using a CellTiter-Blue kit (Promega). For *P311 *adenovirus, cells were infected in PM for 3 days (3I), followed by 3 day DM (3I 3D), or by 6 days in DM (3I 6D), or 10 days in DM (3I 10D). Stably transfected cells were grown in PM for 3 days (PM), followed by 3 day in DM (DM 3D), or 6 days in DM (DM 6D), or 10 days in DM (DM 10D). Results expressed as mean ± standard deviation from triplicate samples within the same experiment.

To assess the function of P311 in muscle cells, we over-expressed *P311 *in C2C12 muscle cells by stable transfection as well as by infection with a *P311*-adenovirus construct (Fig. 7). Expression of both constructs could be demonstrated by *P311 *mRNA detection (Fig. 7A), and by P311-FLAG fusion protein detection by immunofluorescence (Fig. 7B). Cell cytotoxicity assays that measured metabolic capacity showed no significant difference between infected/transfected cells, and control cells over an extended period of 10 days differentiation, which indicated similar cell viability (Fig. 7C). This finding also indicates that over-expression of *P311 *is not detrimental to C2C12 cells.

### P311 over-expression increased C2C12 cell proliferation and reduced differentiation

**Figure 8:**
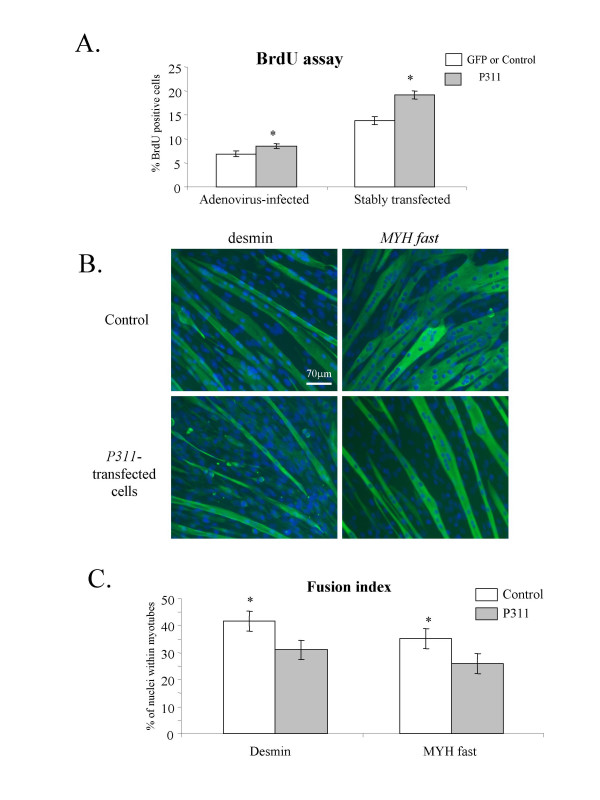
**Effects of P311 on C2C12 cell proliferation and differentiation**. (**A**) BrdU assay was performed on *P311*-infected and adenovirus-GFP control cells, as well as *P311*-transfected and vector only-transfected cells. For both approaches, *P311 *over-expression significantly increased cell proliferation. (**B**)*P311*-stably transfected C2C12 myotubes (differentiated for 9 days) were separately immunostained for desmin and MYH fast, along with DAPI nuclei staining. Under immunofluorescence, myotubes appeared less abundant in *P311*-transfected cells. (**C**) Fusion index quantification on immunostained images showed that *P311*-transfected cells resulted in less mytoube formation than control cells. Asterisks indicate statistical significance. BrdU assay and fusion index determination were analysed with SAS software, differences between pair-wise combinations of the least square means were tested for significance (*p < 0.05). Error bars were standard error, n = 3 replicates from 3 independent experiments.

To determine the effect of *P311 *on cell proliferation, 5'-bromo-2'-deoxyuridine (BrdU) assays were performed on *P311*-adenovirus infected and stably transfected C2C12 cells. Both constructs promoted proliferation compared with control GFP cells (Fig. 8A). C2C12 cells stably transfected with *P311 *gave rise to less myotubes as visualised by immunostaining for desmin, and MYH fast proteins (Fig. 8B). This observation could be appreciated quantitatively by determining the fusion index of the transfected myotubes (Fig. 8C), which showed that *P311 *over-expression produced less myotube formation than control.

**Figure 9:**
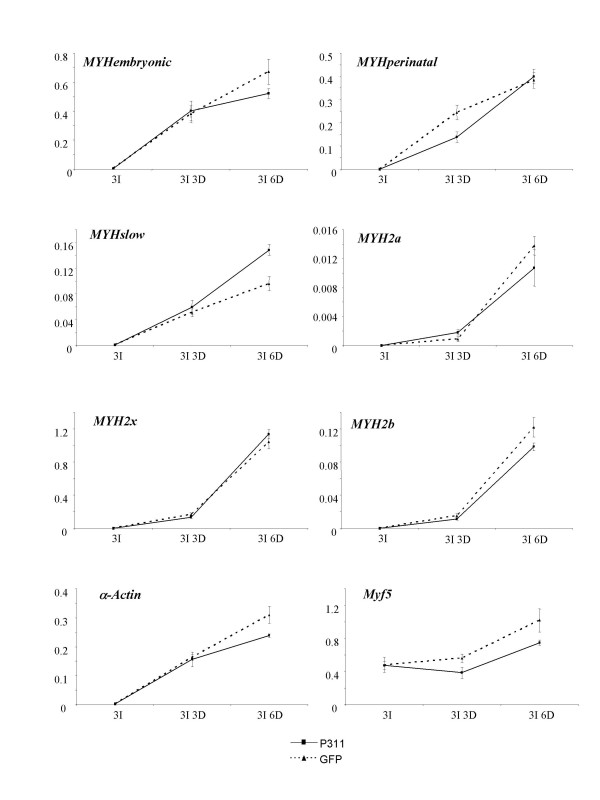
**Muscle gene expression in *P311*-adenovirus infected C2C12 cells**. Real-time PCR was performed on cDNAs derived from infected cells over a time course as detailed in Fig. 4. No dramatic difference in *MYH *expression between *P311*-infected and control C2C12 cells was seen during the early infection time points (3I and 3I 3D). Reduction in expression of *MYHembryonic, MYH2b, Myf-5 *and *α-actin *in *P311*-infected C2C12 myotubes was noticeable at the last time point (3I 6D). Results expressed as mean ± standard deviation from triplicate samples within the same experiment.

**Figure 10:**
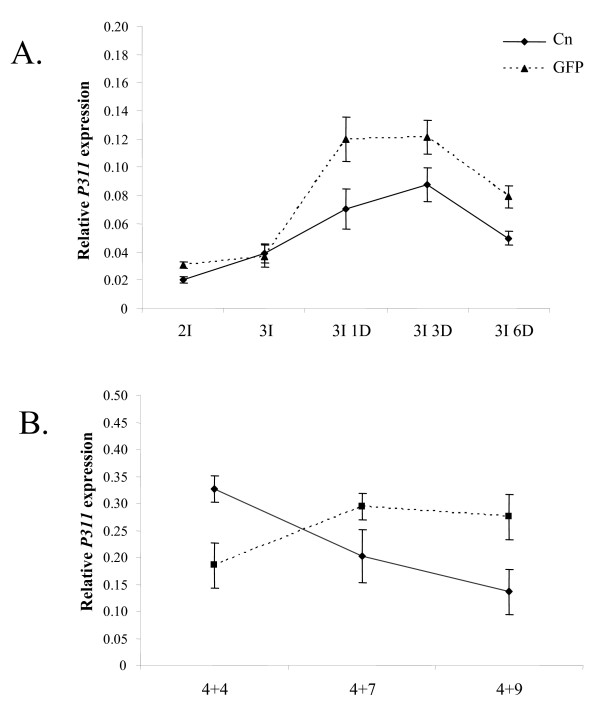
**Effect of calcineurin over-expression on endogenous *P311 *expression in C2C12 cells**. (**A**) Cells were infected with a constitutively active calcineurin (Cn) or GFP-control adenoviral construct in PM for 2 days (2I), or for 3 days (3I), followed by 1 day DM (3I 1D), or 3 days in DM (3I 3D), or 6 days in DM (3I 6D). (**B**) Cells were also infected as myotubes (at 4 days in DM) for 2 days, followed by a further 2 days in DM (4+4), or 5 days in DM (4+7), or 7 days in DM (4+9). In both series (A and B), extended expression of Cn led to a reduction of P311 expression. Results expressed as mean ± standard deviation from triplicate samples within the same experiment.

No obvious differences in *MYH *expression were detected between *P311*-infected and control C2C12 cells during the early stages (3I and 3I 3D) of development (Fig 9). However, at the later stage of differentiation (3I 6D), there was the suggestion of reduced expression of *MYHembryonic, MYH2b, Myf-5 *and *α-actin *in *P311*-infected C2C12 myotubes (Fig. 9), with the notable exception of *MYHslow *which showed raised expression. The overall results of extended *P311 *over-expression indicated a trend towards reduction in the expression of a number regulatory and structural muscle genes, which was consistent with reduced muscle differentiation. Since *P311 *appeared to associate with increased proliferation (Fig. 8A), and reduced differentiation and myotube formation (Fig. 8B to 9), we examined whether it might also be down-regulated by the over-expression of constitutively active calcineurin (Cn), a key mediator of skeletal muscle differentiation [20,21]. Indeed, endogenous expression of *P311 *was down-regulated a few days after Cn infection, regardless of the developmental stage of the C2C12 cells (Fig. 10). Hence, in the promotion of muscle differentiation, Cn down-regulated *P311 *expression.

## Discussion

### PCS is associated with extensive muscle fibre atrophy

PCS is a well-recognised, commercially important clinical congenital condition of piglets. However, woefully little is known about its aetiology, pathogenesis or pathology. In this study, we demonstrated that PCS muscles (LD, ST and G) consistently showed fibre atrophy with concomitant increase in fibre density (Fig. 1B). It appears that PCS is an extensive muscle condition that affects several muscle groups. Indeed, previous work has shown that PCS affects different muscles [1,15]. We found that the ST and LD were more severely affected than the G (Fig. 2A). PCS associated atrophy was not accompanied by significant changes in fibre type composition (Fig. 2B), but some affected muscles showed significant reduction in the expression of *MYHslow*, *MYH2x *or *MYH2b *gene (Fig. 3). Hence PCS is a condition associated with muscle fibre atrophy ostensibly connected to reduced protein accretion, and is associated with reduction in the expression of structural muscle genes. At present, it is unclear if the atrophic fibre change observed in PCS muscles is a primary pathology of the condition or is a secondary outcome of disuse atrophy. In the present work, due to technical difficulty in obtaining complete cryostat cross-sections representative of total muscle areas, total fibre number of each muscle could not be reliably determined. At present, it is not certain if PCS-associated fibre atrophy is also accompanied by fibre hypoplasia.

### PCS is associated with muscle wasting

MAFbx, an E3 ubiquitin ligase enzyme, was identified as an early marker of atrophy through differential expression screening studies in multiple models of skeletal muscle atrophy [16,17]. E3 ubiquitin ligase is one of three enzymatic components of the ubiquitin-proteasome pathway [22], a major protein degradation route known to be responsible for skeletal muscle atrophy [23]. We demonstrated high levels of *MAFbx *mRNA in all 4 atrophic PCS piglets from 4 litters (presented as individual and combined litters) (Fig. 4), in contrast with basal levels found in normal muscles pre- and post-natally (Fig. 6A). The elevated expression of *MAFbx *would suggest that the PCS muscles were subjected to a process of muscle wasting, possibly as a consequence of disuse atrophy. It remains possible that PCS is primary condition of gestational muscle under development.

### Functional significance of P311

P311, first identified in murine embryonic neurons [24], is a small 8 kDa 68-amino acid protein with a short half-life of around 5 minutes [25]. It is characterised by the presence of a conserved PEST domain (sequences rich in proline, glutamic acid, serine and threonine) a targeted site for degradation by the ubiquitin-proteasome system [26]. We found rising levels of *P311 *expression throughout gestation, which was maintained in post-natal normal muscles (Fig. 6B). By contrast, in PCS muscles, *P311 *was detected at much reduced levels (Fig. 5 and 6B). Expression of *P311 *was previously reported to be down-regulated in atrophic murine muscles [17]. In conditions of muscle wasting, arising from denervation or disuse, the process of protein degradation exceeds the rate of protein synthesis, resulting in net protein loss [17]. In PCS muscles, reduced *P311 *expression could be a consequence of net protein loss. On the other hand, given that P311 promoted cell proliferation (Fig. 8A), it may have an active role in promoting muscle growth through raised myoblast number.

P311 does not belong to any known family of proteins, and its cellular function remains largely unclear. To investigate the function of *P311 *in skeletal muscle, it is necessary to ascertain its effects in muscle in the context of cell proliferation, differentiation and phenotype determination. We established that *P311 *over-expression led to raised C2C12 cell proliferation and reduced myotube formation (Fig. 8A and 8C). Consistent with reduced myotube formation, expression of several muscle genes (*MYHembryonic*, *MYH2b*, *α-actin *and *myf-5*) was down-regulated in late differentiation of *P311*-over-expressed C2C12 cells (Fig. 9). Myf-5, like MyoD, is transcriptionally active in proliferating myoblasts; its exogenous expression can cause non-myogenic cells to differentiate and fuse into myotubes [27]. Previously, P311 was shown to be involved in glioblastoma cell migration and fibroblast cell proliferation [28,29]. Moreover, differentiation of neural cells was related to loss of *P311 *expression [25]. Hence, *P311 *may play an active part in the determination of muscle mass through the promotion of myoblast proliferation. The endogenous expression of *P311 *was suppressed by Cn over-expression (Fig. 10). As Cn is a key mediator of muscle differentiation [20,21] it would suggest that P311 could also have an effect of limiting muscle differentiation. In PCS muscles, reduced *P311 *expression could conceivably mediate fibre atrophy through reduced cell proliferation, leading to reduced availability of total myoblast number in the formation of muscle fibres. Moreover, reduced myoblast number could potentially lead to the formation of less muscle fibres, hence fibre hypoplasia. Future work will require detailed total fibre counting to determine if fibre hypoplasia is also a feature of PCS muscles.

## Conclusion

In conclusion, we demonstrated that the PCS is a condition characterised by extensive fibre atrophy and raised fibre density with no significant difference in fibre type composition. At the molecular level, PCS muscles showed reduced expression of a number of sarcomeric genes, elevated *MAFbx *and reduced *P311 *expression. It seems likely that the development of clinical PCS is a function of fibre size and, possibly, fibre number at birth, such that below a certain fibre threshold, the newborn would no longer be able to properly support its own weight. The differential expression patterns of *MAFbx *and *P311 *between muscles of normal and affected litter mates indicate their potential for use as biomarkers in the diagnosis of subclinical PCS. Clearly, more work is needed to evaluate this potential.

## Methods

### Immunohistochemistry and image analysis

ST, LD and G muscles were removed from four 2-day-old splayleg male piglets, along with 4 corresponding normal litter mates. Ten μm thick serial sections were immunostained with antibody for β-dystroglycan used at 1:200 dilution (VisionBiosystems), for MYH slow used at 1:50 dilution (M-32, Sigma) and for MYH fast used at 1:50 dilution (NOQ7.5.4D, Sigma), as previously described [30]. For each muscle sample, 6 fields with at least 500 fibres each were randomly selected for morphometric analysis under × 20 magnification using the KS300 image analysis software (Carl Zeiss Ltd).

### Expression constructs

Full length porcine *P311 *cDNA and a constitutively active murine calcineurin A (Cn) (from Dr. S. Williams, University of Texas) were cloned into an adenovirus vector, using the Adeno-X Expression System 2 (BD Biosciences). The starting plasmid vector pDNR-CMV for the insertion of *P311 *and Cn housed an expression cassette that was derived from the plasmid pAAV-IRES-hrGFP (Stratagene) which comprised a multiple cloning site for the creation of a 3'-end FLAG fusion gene and a GFP reporter gene, with an internal ribosomal entry site (IRES) between the two. For time course studies, infection with *P311 *and Cn-adenovirus constructs were used at multiplicity of infection (MOI) of 5. For stable transfection, full length *P311 *cDNA was cloned into a neomycin resistant pBK-CMV expression plasmid vector (Stratagene), where transcription was driven by a cytomegalovirus immediate early promoter.

### Cell culture, transfections and expression assays

C2C12 cells (CRL-1772) were grown in proliferation medium (PM) (10% foetal bovine serum in DMEM with 100 units/ml penicillin and 100 μg/ml streptomycin). At 80% confluence, PM was replaced by differentiation medium (DM) (4% horse serum in DMEM with penicillin and streptomycin). For stable transfections, C2C12 cells at 35% confluence in T25 culture flasks were transfected with 6.0 μg *P311 *plasmid, by the use of lipofectamine and Opti-MEM, according to manufacturer's instructions (Invitrogen). Next day, the transfection medium was replaced with PM for 1 day, followed by G418 (Invitrogen) selection at 2000 μg/ml over several days until a day after all cells in the control untransfected flask had been eliminated by G418. All subsequent experiments on stably transfected cells were conducted in the absence of G418.

### Immunofluorescence

C2C12 cells, infected with *P311 *or GFP control adenovirus, were grown for 3 days in PM, followed by 8 days in DM. To improve P311 stability, o-phenanthorline and lactacystin were added to the DM, at final concentrations of 1.26 mM and 10 mM respectively, 2 hour prior to fixation. Cells were fixed in 4% paraformaldehyde and incubated with the primary anti-FLAG M2 monoclonal antibody (Sigma), at a dilution of 1/2000, overnight at 4°C. A goat anti-mouse-TRITC (Southern Biotech) at 1/200 was used as the secondary antibody in a 45 minute incubation at 37°C. It was mounted using Vectashield (Vector) hard set mounting medium with DAPI. Images were captured by a CCD camera mounted on an inverted Olympus fluorescence microscope.

### BrdU, fusion index and cell cytotoxicity assays

BrdU assays on C2C12 cells, infected with *P311*-adenovirus and stably transfected with *P311 *plasmid construct, were performed as previously described [31]. Briefly, cells were incubated with BrdU for 2 hours and immunostained with a rat anti-BrdU monoclonal antibody. BrdU positive cells were counted. For each construct, three independent experiments were conducted.

For fusion index assays, after 9 days in DM, cells were fixed and incubated with a primary antibody (anti-desmin 1:200, Dako, or anti-MYH fast 1:200) at 4°C overnight. Secondary FITC conjugated sheep anti-mouse antibody (Sigma) was used at 1/200 dilution. For each sample, 5 fields were randomly chosen for counting. Fusion index was defined as the number of DAPI stained nuclei within myotubes in a given field divided by the total number of DAPI stained nuclei in the same field. Three independent experiments were conducted.

Cell cytotoxicity assay, a resazurin-based reduction assay that measured the metabolic capacity of cells, was performed according to manufactururer's protocol, normalised to unit weight of protein (CellTiter-Blue, Promega).

### Quantitative real-time RT-PCR

Total RNA was isolated from porcine muscles and C2C12 cells using the RNeasy Mini kit (Qiagen), quantitated in an Agilent 2100 bioanalyzer and converted into cDNA with random primers using a Reverse Transcription System (Promega). Two μg of total RNA were used for each cDNA conversion. Four sets of cDNA samples from PCS and normal piglets were prepared (litters 1 to litters 4). With pre-natal muscles, LD was pooled from 3 Pietrain-based pig foetuses from gestation stage of 35, 49, 63, 77 and 91 days [32]. Additionally, pooled whole 14-day embryos and 21-day embryos derived from the same uterine horn were used [32]. With *P311 *adenovirus, C2C12 cells were infected in PM for 3 days (3I), followed by 3 days in DM (3I 3D) or by 6 days in DM (3I 6D). TaqMan quantitative real-time RT-PCR (Applied Biosystems) was performed on a number porcine and murine genes: *MYHembryonic*, *MYHperinatal*, *MYHslow*, *MYH2a*, *MYH2x*, *MYH2b*, β-*actin*, α *-actin*, *myf-5, MAFbx *and *P311*. Sequence details of primers and TaqMan probes of porcine genes were previously given [18]. All other primers and probes sequences are shown in Table 1. A relative standard curve method, normalised to β-actin was used in the quantification of expression.

### Statistical analyses

SAS software (SAS procedure mixed, SAS Institute) was used. Fibre size, fibre density, real-time PCR data for PCS (Fig. 3 to 5) were analyzed by mixed model variance analysis using muscle type (ST, LD, and G), disease status (normal, PCS) as fixed effects with litters and replicate as random effects. BrdU and fusion index data were analyzed by mixed model variance analysis, using treatment (*P311 *and GFP control) as fixed effects with experiments and replicate as random effects. The standard errors of the difference between sample means were calculated using the error mean square from the ANOVA. Differences between pair-wise combinations of the least square means were tested for significance (p < 0.05). Unless specifically stated, quantitative real-time RT-PCR data were expressed as mean ± standard deviation from triplicate samples within the same experiment.

## Authors' contributions

PTO performed most of the experimental work and wrote the draft manuscript. NDC helped with the real time RT-PCR experiments and provided intellectual input to the work. JE helped with the cell culture procedures and project planning. KCC initiated and coordinated the study, and critically revised the manuscript. All authors read and approved the final manuscript.

## Figures and Tables

**Table 1:** Sequences of primers and TaqMan probes

**Gene**	**Primer**	**Sequence 5' → 3'**
Porcine *MAFbx*	S	AAGCGCTTCCTGGATGAGAA
	A	GGCCGCAACATCATAGTTCA
	P	AGCGACCTCAGCAGTTACTGCAACAAGG
Porcine *P311*	S	GAG GGA AGG CCT AAG GG
	A	CGG TCT CGC CAT CCT TCT T
	P	ACT TCC CAT CCC AAA GGA AGT GAA CCG
Murine *P311*	S	CAGCCAAGAACCGTTTGCAT
	A	CACTTCCTTAGGCACGGGAA
	P	TTCCCTTAATAAGACCTCCCTCCATTTCCTTG
Murine *MYHslow*	S	GCC TGG GCT TAC CTC TCT ATC AC
	A	CTT CTC AGA CTT CCG CAG GAA
	P	CGT TTG AGA ATC CAA GGC TCA
Murine *MYH2a*	S	CAG CTG CAC CTT CTC GTT TG
	A	CCC GAA AAC GGC CAT CT
	P	TGA GTT CAG CAG TCA TGA G
Murine *MYH2x*	S	GGA CCC ACG GTC GAA GTT G
	A	GGC TGC GGG CTA TTG GTT
	P	CTA AAG GCA GGC TCT CTC ACT GGG CTG
Murine *MYH2b*	S	CAA TCA GGA ACC TTC GGA ACA C
	A	GTC CTG GCC TCT GAG AGC AT
	P	TGC TGA AGG ACA CAC AGC TGC ACC T
Murine *Myf5*	S	CAG CCC CAC CTC CAA CTG
	A	GCA GCA CAT GCA TTT GAT ACA TC
	P	TGT CTG GTC CCG AAA GAA CAG CAG CTT
Murine *MYHembryonic*	S	TCC GAC AAC GCC TAC CAG TT
	A	CCC GGA TTC TCC GGT GAT
	P	ATG CTG ACT GAT CGT GAG AAC CAG TCT ATC CT
Murine *MYHperinatal*	S	GGA GGC CAG GGT ACG TGA A
	A	GAG CAC ATT CTT GCG GTC TTC
	P	AGG AAC TTA CCT ACC AGA CTG
Murine *α-actin*	S	GAG CGT GGC TAT TCC TTC GT
	A	CAC ATA GCA CAG CTT CTC TTT GAT
	P	CGC GCA CAA TCT CAC GTT CAG CTG
Murine *β-actin*	S	CGT GAA AAG ATG ACC CAG ATC A
	A	CAC AGC CTG GAT GGC TAC GT
	P	TTG AGA CCT TCA ACA CCC CAG CCA TG

S, sense; A, antisense; P, TaqMan probe. All probes were 5' labelled with FAM, and 3' labelled with TAMRA.
